# Down Regulation of SIRT2 Reduced ASS Induced NSCLC Apoptosis Through the Release of Autophagy Components *via* Exosomes

**DOI:** 10.3389/fcell.2020.601953

**Published:** 2020-12-03

**Authors:** Lei Wang, Pei Xu, Xiao Xie, Fengqing Hu, Lianyong Jiang, Rui Hu, Fangbao Ding, Haibo Xiao, Huijun Zhang

**Affiliations:** ^1^Department of Cardiothoracic Surgery, Xinhua Hospital Affiliated to Shanghai Jiao Tong University School of Medicine, Shanghai, China; ^2^Department of Cardiothoracic Surgery, Huashan Hospital of Fudan University, Shanghai, China

**Keywords:** acute shear stress, autophagy, exosome, SIRT2, TFEB, NSCLC, metastasis

## Abstract

Metastasis of cancer is the main cause of death in many types of cancer. Acute shear stress (ASS) is an important part of tumor micro-environment, it plays a crucial role in tumor invasion and spread. However, less is known about the role of ASS in tumorigenesis and metastasis of NSCLC. In this study, NSCLC cells were exposed to ASS (10 dyn/cm^2^) to explore the effect of ASS in regulation of autophagy and exosome mediated cell survival. Finally, the influence of SIRT2 on NSCLC cell metastasis was verified *in vivo*. Our data demonstrates that ASS promotes exosome and autophagy components releasing in a time dependent manner, inhibition of exosome release exacerbates ASS induced NSCLC cell apoptosis. Furthermore, we identified that this function was regulated by sirtuin 2 (SIRT2). And, RNA immunoprecipitation (RIP) assay suggested SIRT2 directly bound to the 3′UTR of transcription factor EB (TFEB) and facilitated its mRNA stability. TFEB is a key transcription factor involved in the regulation of many lysosome related genes and plays a critical role in the fusion of autophagosome and lysosome. Altogether, this data revealed that SIRT2 is a mechanical sensitive protein, and it regulates ASS induced cell apoptosis by modulating the release of exosomes and autophagy components, which provides a promising strategy for the treatment of NSCLCs.

## Introduction

Lung cancer stands as the leading cause of cancer-related deaths world-wide (Siegel et al., 2019). Non-small lung cancer (NSCLC) accounts for 87% of all lung cancers, with about 75% of cases diagnosed at a later stage leading to poor prognosis (Cancer Genome Atlas Research Network, 2014). Radiation and chemotherapy are valid available treatment strategies, but these approaches cannot improve the poor 5 years survival rate of these patients, which ranges from 5 to 31% (Herbst et al., 2018). Metastasis is the key contributor to tumorigenesis in many cancers, including NSCLC (Gassmann et al., 2004; Gupta and Massague, 2006). Metastasis is defined as the migration of cancer cells from its primary site of origin to progressively invade and colonize the neighboring tissues (Steeg, 2016). Cancerous cells migrate either by entering the blood stream directly or through the lymphatic system (Chaffer and Weinberg, 2011). Several proangiogenic factors secreted by the tumor cells aid in the formation and connection with the capillary system which allows intravasation and circulation of these cells in the blood stream (Folkman, 1986; Sitohy et al., 2012). Once in the blood stream, cells encounter multiple stresses due to flow of blood, lymph or other tissue fluids (Tarbell et al., 2014). Shear stress (SS) is a key factor that has previously been associated with organogenesis and maintenance (Rutkowski and Swartz, 2007). In spite of the negative effects of SS, studies have indicated low-shear stress enhances migration, proliferation, and differentiation of circulating tumor cells (CTCs) (Sebastian et al., 2020). Studies have attempted to elucidate how CTCs can survive in such harsh conditions, and it has been suggested that they undergo multiple morphological and molecular changes to adapt and survive under mechanically stressful conditions (Cognart et al., 2020). Interestingly, it has been reported that SS can induce autophagy in different cancer cell types (Fleury et al., 2006; Das et al., 2018). Autophagy has varying roles in multiple cell types, critically cancer cells depend on autophagy to maintain a minimal metabolic requirement and survive under harsh conditions through self-degradation of organelles and protein aggregates (Mowers et al., 2018). In an *in vitro* study by Lien et al. (2013) found that SS (0.5, 6, and 12 dyn/cm^2^) increased autophagy which was evident from the acidic vesicular organelle formation, microtubule-associated protein light chain 3 (LC3B) transformation and p62/SQSTM1 degradation in HCC cells. In addition, to protect cells against these autophagic components, cancer cells under acute SS have an alternative protection mechanism through the release of autophagic components through extracellular vesicle (EV) release (Reck et al., 2014). Recently, a study has indicated such release of exosomes are regulated by a member of the sirtuin family, SIRT1. A study by Latifkar et al. (2019), indicated that SIRT1 down-regulation altered the secretome and increased the exosome release in breast cancer cell lines. This study further indicated that SIRT1 reduced the tumorigenesis of breast cancer by altering the lysosomal activity. However, sirtuin’s role in NSCLC is still not clear.

The aim of this study is to assess the effect of ASS in NSCLC and to elucidate the role of sirtuins in stress response and survival of cancer cells. Initially, we identified that NSCLCs exposed to ASS (10 dyn/cm^2^) clearly shed increased level of exosomes and autophagic components. Interestingly, ASS exposed NSCLCs expressed less SIRT2 and the expression of SIRT1 remained unaffected. Further, it was evident that SIRT2 regulated autophagosome formation, apoptosis, and metastasis by binding and regulating the expression of a key player in the lysosomal biogenesis and autophagy, transcription factor EB (TFEB). Thus, this study aims to elucidate the role SIRT2 and TFEB in NSCLC tumorigenesis and shed light into ASS associated mechanisms, thus aiding in identification of potential target for treatment strategies against NSCLC.

## Materials and Methods

### Clinical Samples

We collected forty pairs of NSCLC tissue samples and healthy lung tissues from the Xin Hua Hospital Affiliated to Shanghai Jiao Tong University School of Medicine. Based on the TNM criteria for NSCLC, we grouped and selected samples for this study. Patients who previously received chemotherapy or radiotherapy were eliminated from the study. All subjects provided written informed consents. This study was approved by the Institutional Review Board of Xin Hua Hospital Affiliated to Shanghai Jiao Tong University School of Medicine and is in compliance with the principles laid down in the Declaration of Helsinki.

### Cell Culture

NSCLC cells A549 and H1299 were purchased from American Type Culture Collection (ATCC, Manassas, VI, United States). The cells were cultured in Dulbecco’s Eagle Medium (DMEM) with 10% fetal bovine serum (FBS) and 1% penicillin/streptomycin (Invitrogen, Carlsbad, CA) in 37°C and 5% CO_2_.

### Plasmids and Lentivirus

The pcDNA3.1 vector (Invitrogen, United States) with a full-length cDNA sequence of either SIRT2 or TFEB were used to overexpress SIRT2 or TFEB, respectively. Empty pcDNA3.1 vector and scrambled shRNA or siRNA were used as negative controls. Further, Lipofectamine^TM^ 2000 (Invitrogen, United States) was used to transfect H1299 or A549 cells with the above-mentioned plasmids. Post 48 h of transfection, cells were collected for further use.

### Ad-mCherry-GFP-LC3B Transfection

A549 and H1299 cells were seeded onto 24 well plate and cultured in DMEM complete medium till 50% confluence. Further, the complete medium was replaced with viral solution with MOI of 10. Post 24 h, the medium was removed and the cells were washed with PBS and DMEM medium. Further, the cells were continued to be cultured in DMEM medium till use.

### Quantitative Real-Time PCR (RT-qPCR)

Total RNA was isolated using the Trizol reagent (Gibco, Carlsbad, CA, United States). Using primescript RT reagent kit, the RNA was reverse transcribed to cDNA, and SYBR-Green qPCR (Invitrogen) was performed using an Applied Biosystems system (Foster City, CA, United States). The primers used for these experiments were SIRT2 Forward: 5′-TGCGGAACT TATTCTCCCAGA-3′; Reverse: 5′-CCAGCCGATACTCGT TCAGC-3′, TFEB Forward: 5′-ACCTGTCCGAGACCTATGGG- 3′; Reverse: 5′-CGTCCAGACGCATAATGTTGTC-3′, GAPDH Forward: 5′-GGAGCGAGATCCCTCCAAAAT-3′; Reverse: 5′-GGCTGTTGTCATACTTCTCATGG-3′. The relative abundance of mRNA was normalized to GAPDH and the 2^–ΔΔCT^ method was used to analyze expression levels.

### Western Blotting Analysis

Total protein was isolated from tissue samples using RIPA buffer (Sigma-Aldrich). After quantification using Bradford assay(Thermo Fisher Scientific, Waltham, United States), 20 μg of total protein were pretreated with loading buffer and loaded in 5–20% SDS-polyacrylamide gel (Bio-Rad Laboratories, Hercules, United States). After migration, the samples were transferred into a nitrocellulose membrane (GE Healthcare Life Science). Subsequently, the membranes were blocked using 5% skim milk in PBS buffer for 1 h. Further, the membranes were incubated overnight at 4°C in the primary antibody diluted in 1% skim milk in PBS buffer. The primary antibodies used were anti-SIRT2 antibody (Cell Signaling Technology, Beverly, CA), anti-TFEB antibody (Abcam, Cambridge, United Kingdom), anti-TSG101 antibody (Abcam, Cambridge, United Kingdom), anti-CD63 antibody (Abcam, Cambridge, United Kingdom), anti-HSP70 antibody (Abcam, Cambridge, United Kingdom), and anti-LC3 antibody (Cell Signaling Technology, Beverly, CA). For housekeeping, anti-β-actin antibody (Sigma, St. Louis, MO) was used. After washes with PBS, the membranes were incubated with HRP-conjugated secondary antibodies (Cell Signaling Technology, Danvers, MA) and were visualized using Odyssey Imaging Systems (LI-COR Biotechnology, Lincoln, United Kingdom).

### TUNEL

Using TUNEL detection kit (Beyotime, Hangzhou, China), apoptosis in A549 and H1299 cells were monitored following the manufacturer’s protocol. Cells cultured in a 24 well dish were initially incubated with proteinase K followed by TUNEL reaction mixture for 1 h at 37°C. Further, the cells were monitored using a confocal microscope (Leica, Germany). Controls were incubated with DNase I (positive control) or labeling solution (negative control) for 1 h. Percentage of TUNEL positive cells were assessed using MetaMorph image 7.1.2.0 software.

### Isolation of Exosomes

Initially, 10% FBS was centrifuged overnight at 100,000 g to remove any existing exosomes. Further, the cells were cultured in medium with the above mentioned FBS for 24 h and the medium was centrifuged at 320 g for 10 min followed by centrifugation at 2,000 g for 15 min which allows removal of any cellular debris. Further, the supernatant was again centrifuged 10,000 *g* for 30 min at 4°C. The supernatant collected from the previous step was again centrifuged at 100,000 *g* for 70 min and the pellet was washed with PBS and recentrifuged at 100,000 *g* for 70 min. The pellet contained exosomes which was further processed for other experiments.

### Transmission Electron Microscopy (TEM)

When NSCLC cells (H1299 and A549 cells) reached 90% confluence, cells were collected and centrifuged at 1,500 rpm for 15 min. To the pellet, 0.5% glutaraldehyde fixative was added and incubated for 10 min at 4°C. Further, the samples were centrifuged at 12,000 rpm for 12 min. After discarding the supernatant, 3% glutaraldehyde was added. Finally, 1% osmium tetroxide was used to fix again, dehydration was performed using acetone, and embedding was done using Epson812. Ultra-thin sections were obtained and double staining with uranyl acetate and lead citrate was performed. Finally, exosomes and autophagosomes were observed using TEM.

### Nanoparticle Tracking Analysis (NTA)

The isolated exosomes were assessed with NanoSight NS300 (Malvern, Cornell NanoScale Science and Technology Facility) to determine the sizes and concentrations of exosomes in a given sample. Further, the data was assessed using NTA 3.2 Dev Build 3.2.16.

### Flow Cytometry of Apoptosis

Annexin V-FITC Apoptosis Detection Kit (Sigma-Aldrich) was used to assess apoptosis rate after exposure to ASS. Cells were suspended in binding buffer and incubated at 37°C. Further, the cells were incubated with 10 μL Annexin V-FITC and 5 μL PI in the dark. Finally, cell apoptosis rate was measured by Flow Cytometer using FlowJo software (Beckman Coulter, Fullerton, CA, United States).

### *In vitro* Invasion Assay

4 × 10^4^ cells were suspended in 200 μL of serum-free medium, and seeded into an upper chamber of the Transwell chamber (8 μm pore size; Millipore, Burlington, MA, United States) which was pre-coated with 20 μg Matrigel (BD Biosciences) while medium supplemented with 10% FBS was added into the lower chamber after 24 h incubation at 37°C. The non-invaded cells in the upper chamber were removed, and the cells that invaded the matrix were fixed with 4% paraformaldehyde for 20 min and stained with 0.5% crystal violet. Finally, the invaded cells are counted under the microscope.

### Immunofluorescence Staining (IFC)

Cells were initially washed with PBS and fixed with 4% paraformaldehyde for 10 min. Further, after thorough washing with PBS, the cells were blocked with 1% BSA in PBST (PBS + 0.1% Tween 20) for 30 min. Then, the cells were incubated in primary antibody in 1% BSA in PBST overnight (4°C). The primary antibodies used were anti-LC3B antibody (Cell Signaling Technology, Beverly, CA), anti-LAMP1 antibody (Cell Signaling Technology, Beverly, CA), and anti-TFEB antibody (Abcam, Cambridge, United Kingdom). Further after washes with PBST, the cells were incubated with FITC-conjugated or CY3-conjugated secondary antibodies (Abcam, Cambridge, United Kingdom) for 1 h. The cells were finally incubated with DAPI and imaged using an Olympus microscope at 40× magnification. Images were captured using an Olympus FSX100 microscope and analyzed using Image J software.

### RNA Immunoprecipitation (RIP) Assay

After preparing whole-cell lysates from confluent A549 and H1299 cells, equal amounts of lysates and antibodies against SIRT2 or control IgG were loaded onto magnetic beads followed by extensive washing. We performed individual pulldowns at 4°C. Then, we amplified recovered RNA for immunoprecipitation and generated cDNA. The obtained cDNA were used to perform PCR with primers TFEB Forward: 5′-ACC TGTCCGAGACCTATGGG-3′; Reverse: 5′-CGTCCAGACGC ATAATGTTGTC-3′, GAPDH Forward: 5′-GGAGCGAGATC CCTCCAAAAT-3′; Reverse: 5′-GGCTGTTGTCATACTTCTC ATGG-3′. The PCR product was migrated in an agarose gel (1.5%, Biowest, Nuaille, France) and the product was observed using a FluorChem^TM^ Q system (Alpha Innotech, San Jose, CA, United States) and visualized.

### Model of Metastases of NSCLC in Nude Mice

BALB/c nude mice were purchased from Shanghai SLAC Laboratory Animal, aged at 4–6 weeks. All animal experiments included in this study were approved by the Ethical Committee for Animal Research of Xin Hua Hospital Affiliated to Shanghai Jiao Tong University School of Medicine. Further, All animal care procedures accorded with institutional and international guidelines. The mice were housed in a controlled temperature of 22 ± 1°C with a 12 h light/12 h dark cycle with lights on from 0600 to 1,800 h. A549 and H1299 cells were cultured as previously mentioned and a suspension of 1 × 10^6^ cells were injected intraperitoneally into the nude mice (*n* = 6 for each cell type). The mice were weighed once every 3 days for 33 days. After 33 days, the mice were sacrificed and lungs and liver were isolated to assess the levels of metastasis.

### Histological Analysis

Histological analysis was performed using a previously described protocol. Initially tissues were collected and fixed in 10% formalin followed by paraffin embedding and sectioning into 10 μm slices. The sections were stained using hematoxylin-eosin (H&E) and observed and imaged under a light microscope.

### Statistical Analysis

All the data were analyzed using SPSS software. All *in vitro* experiments were repeated at least three times independently. Data are presented as the mean ± standard deviation (SD) unless otherwise stated. *P* < 0.05 was considered significant.

## Results

### ASS Induced NSCLC Cell Autophagy

**FIGURE 1:**
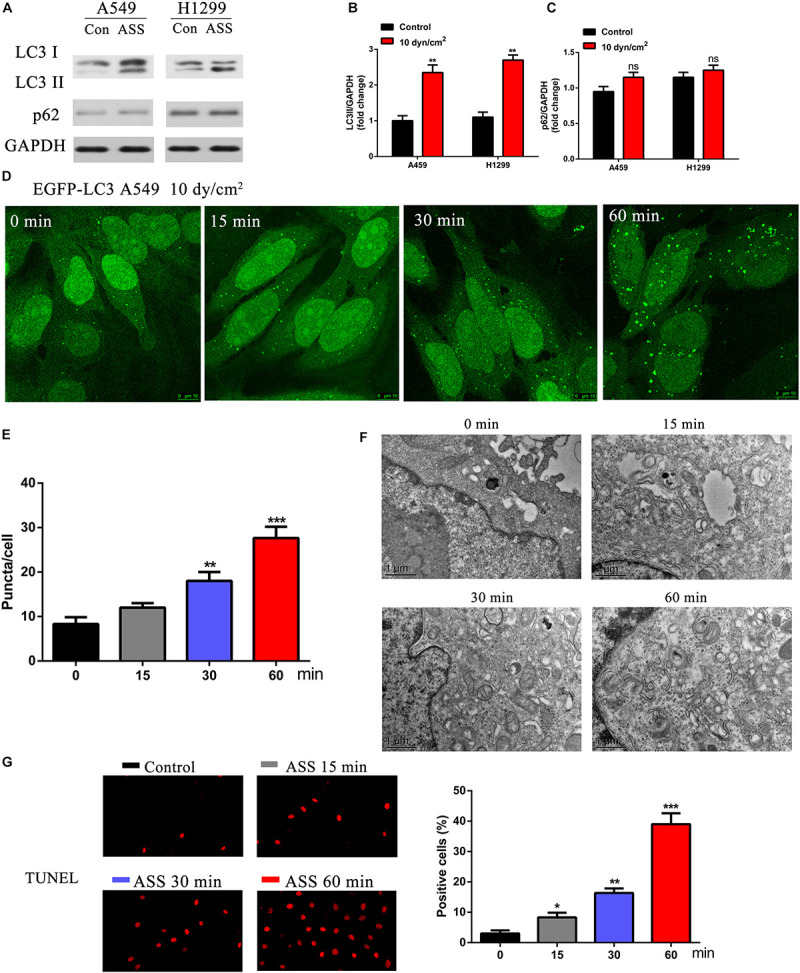
ASS activated NSCLC cell autophagy and promoted cell apoptosis. **(A)** The expression of LC3I/II and p62 were detected by western blot in A549 and H1299 cells treated with ASS (10 dyn/cm^2^) for 60 min or incubated at quiet state. All experiments were repeated three times, **P < 0.01. **(B)** Quantification of LC3II/LC3I ratio in relation to GAPDH, **(C)** Quantification of p62 levels in relation to GAPDH, **(D,E)** A549 cells expressing EGFP-LC3 were exposed to ASS (10 dyn/cm^2^) for indicated times (0, 15, 30, and 60 min); Scale bar, 10 μm. The percentage of cells with EGFP-LC3 puncta were quantified, **P < 0.01, ***P < 0.001. **(F)** TEM images of A549 cells exposed to ASS for 0, 15, 30, and 60 min; Scale bar, 1 μm. **(G)** TUNEL assay was performed after A549 cells were exposed to ASS for indicated times, the percentage of cell apoptosis were quantified by Image Pro Plus software, *P < 0.05, **P < 0.01, ***P < 0.001.

To understand the effects of ASS on NSCLC, two epithelial carcinoma cell lines A549 and H1299 were exposed to ASS (10 dyn/cm^2^) for 60 min. Further, we performed western blotting analysis from the ASS exposed cell lysates and observed that LC3II/GAPDH ratio was significantly increased in the ASS exposed cells compared to the unexposed (maintained in resting state) control cells (Figures 1A–C). Interestingly, there was no significant decrease in the p62 levels in both A549 and H1299 cells. Further, A549 cells expressing EGFP-LC3 (a mammalian ortholog of atg8) were examined. These A549-EGFP-LC3 cells were exposed to ASS (10 dyn/cm^2^) for varied periods of time (0, 15, 30, and 60 min). Evidently, with prolonged exposures to ASS, we could clearly observe a exposure time-dependent increase in the number of EGFP-LC3 green puncta in the cells. By 60 min, a significantly high number of green LC3 puncta could be observed in the ASS exposed cells compared to the cells not exposed to ASS (0 min) (Figures 1D,E). In order to further characterize ASS induced autophagy, A549 cells were exposed to ASS (10 dyn/cm^2^) for varying lengths of time (0, 15, 30, and 60 min), which were further characterized by TEM. TEM has been previously used to trace the process of autophagy, as the autophagosomes can be observed as bi- or multi-layer vacuole like structures encompassing components such as organelles. From the TEM images obtained at different times, it was evident that exposure to ASS increased the formation of autophagosomes in a time-dependent manner. After 60 min of exposure to ASS, it was clear that the cells had increased number of autophagosomes compared to the control (0 min ASS) (Figure 1F). These results indicate that ASS could induce autophagy in A549 cells. Next, to further understand the effect of ASS on apoptosis, we performed TUNEL assay on A549 cells. As shown in Figure 1G, we could observe an increasing number of TUNEL positive cells after 60 min of exposure to ASS. This further indicated a potential role of ASS in apoptosis.

### ASS Promoted Exosomes and Autophagy Related Components Release

**FIGURE 2:**
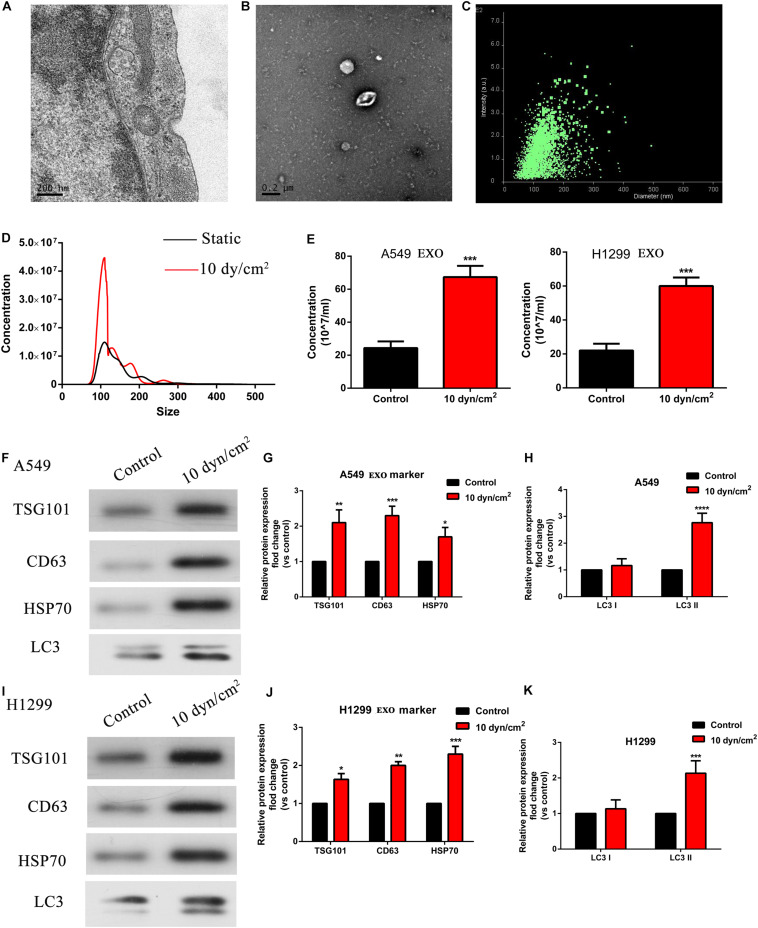
ASS promoted exosomes and autophagy related components release. **(A,B)** A549 cells derived exosomes observed by TEM, Scale bar, 200 nm. **(C)** The intensity and size of exosomes as evaluated by NANOSIGHT. **(D,E)** The concentration of exosomes isolated from A549 and H1299 cells after exposure to ASS for 60 min as acquired by Nanoparticle Tracking Analysis (NTA), ***P < 0.001. **(F–K)** Expression of exosomes maker (TSG101, CD63, and HSP70) and autophagy component maker (LC3I/II) were measured by western blot analysis, every experiment was repeated in triplicate, *P < 0.05, **P < 0.01, ***P < 0.001, ****P < 0.0001.

As recent studies have revealed that mechanical stress affects exosome release of autophagic components, we were interested to observe the effects of ASS on exosomes (Reck et al., 2014). Initially, using TEM, we clearly observed and characterized the A549 cells derived exosomes (Figure 2A). Further, with the aid of a previously published protocol, we isolated the exosomes which were secreted by NSCLC cells; and then confirmed their presence through TEM and NANOSIGHT (Figures 2B,C). Exosomes were also isolated from A549 and H1299 cells exposed to ASS (10 dyn/cm^2^) and the further exosomes characterized with nanoparticle tracking analysis (NTA). It was evident that shared a similar size between control and ASS exposed group (Figure 2D), but the concentration of exosomes were significantly higher in the ASS exposed A549 and H1299 cells (Figure 2E). Expression of exosomes maker (TSG101, CD63, and HSP70) and autophagy component maker (LC3I/II) were assessed by western blot analysis. From the western blot analysis, it was clear that exosomes obtained after exposure to ASS, had high expression levels of TSG101, CD63, and HSP70 (Figures 2F,G,I,J). Interestingly, we also observed high levels of LC3II in the exosomes obtained from both A549 and H1299 after exposure to ASS (Figures 2H,K) compared to the control (static) cell exosomes. These data clearly indicate that ASS promotes exosomes secretion along with release of autophagic components in NSCLC.

### Suppression of Exosomes Release by ASS Will Induce NSCLC Cell Apoptosis

**FIGURE 3:**
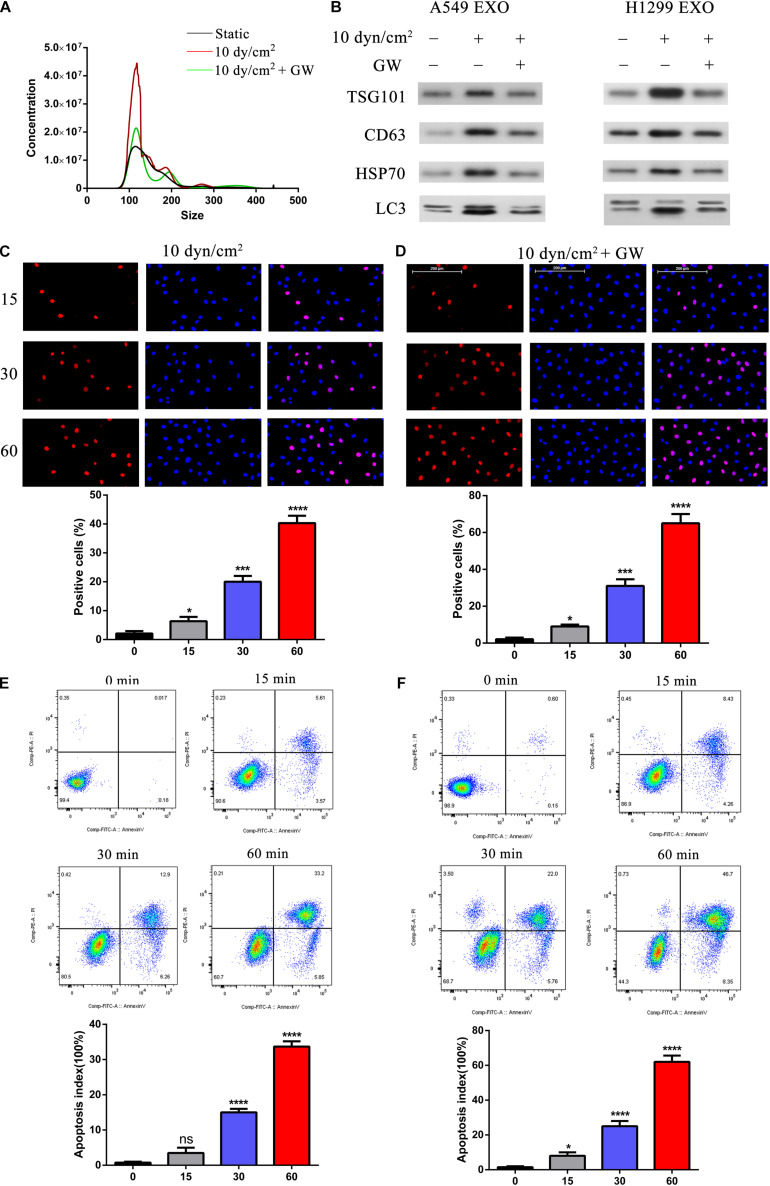
Suppression of exosomes release exacerbated ASS induced NSCLC cell apoptosis. **(A)** A549 and H1299 cells were pretreated with exosome inhibitor GW4689 (GW) for 60 min and then exposed to ASS (10 dyn/cm^2^) for 60 min, the concentration of isolated exosomes were detected by NTA. **(B)** The expression of exosomes marker (TSG101, CD63, and HSP70) and secreted autophagy components (LC3 I/II) were analyzed by western blot. **(C–F)** After pretreatment with GW4689 for 60 min, NSCLC cells were exposed to ASS (10 dyn/cm^2^) for 0, 15, 30, and 60 min, the percentage of cell apoptosis was evaluated by **(C,D)** TUNEL assay and **(E,F)** flow cytometry using Annexin V-fluorescein isothiocyanate (FITC) Apoptosis Staining/Detection Kit, *P < 0.05, ***P < 0.001, ****P < 0.0001.

To understand the relationship between ASS and exosomes, we further treated the NSCLC cell lines with a known exosome inhibitor GW4689 (Essandoh et al., 2015) for 60 min and then exposed these cells to ASS (10 dyn/cm^2^) for 60 min. Exosomes were isolated and assessed by NTA. Interestingly, it was clear that even when cells were exposed to ASS, pretreatment with GW4689 decreased the concentration of exosomes (Figure 3A). Further, western blotting analysis confirmed that exosomes from GW4689 pretreatment group showed significantly decreased exosomes markers (TSG101, CD63, and HSP70) and autophagic marker (LC3II) in both A549 and H1299 cells (Figure 3B). This indicated that GW4689 was successful in inhibiting exosome secretion in ASS exposed NSCLCs. Further to understand the effect of suppression of exosome release on apoptosis of NSCLC, we performed TUNEL assay. Initially A549 cells were pretreated with GW4689 for 60 min and then the cells were exposed to ASS (10 dyn/cm^2^) for 0, 15, 30, and 60 min. By 60 min, in ASS exposed cells, a high number of TUNEL positive cells could be observed (Figure 3C). However, in the GW4689 pretreated cells, by 60 min the number of TUNEL positive cells increased even higher than the ASS exposed group (Figure 3D). This data was further confirmed by flow cytometry using Annexin V-fluorescein isothiocyanate (FITC) Apoptosis Staining/Detection Kit (Figures 3E,F) which indicated that GW4689 pretreatment significantly caused a 30% increase in apoptosis in the ASS exposed cells. Hence, it was clear that ASS increases apoptosis in NSCLC which could be exacerbated by the suppression of exosome secretion.

### Role of SIRT2 on ASS Induced Cell Apoptosis

**FIGURE 4:**
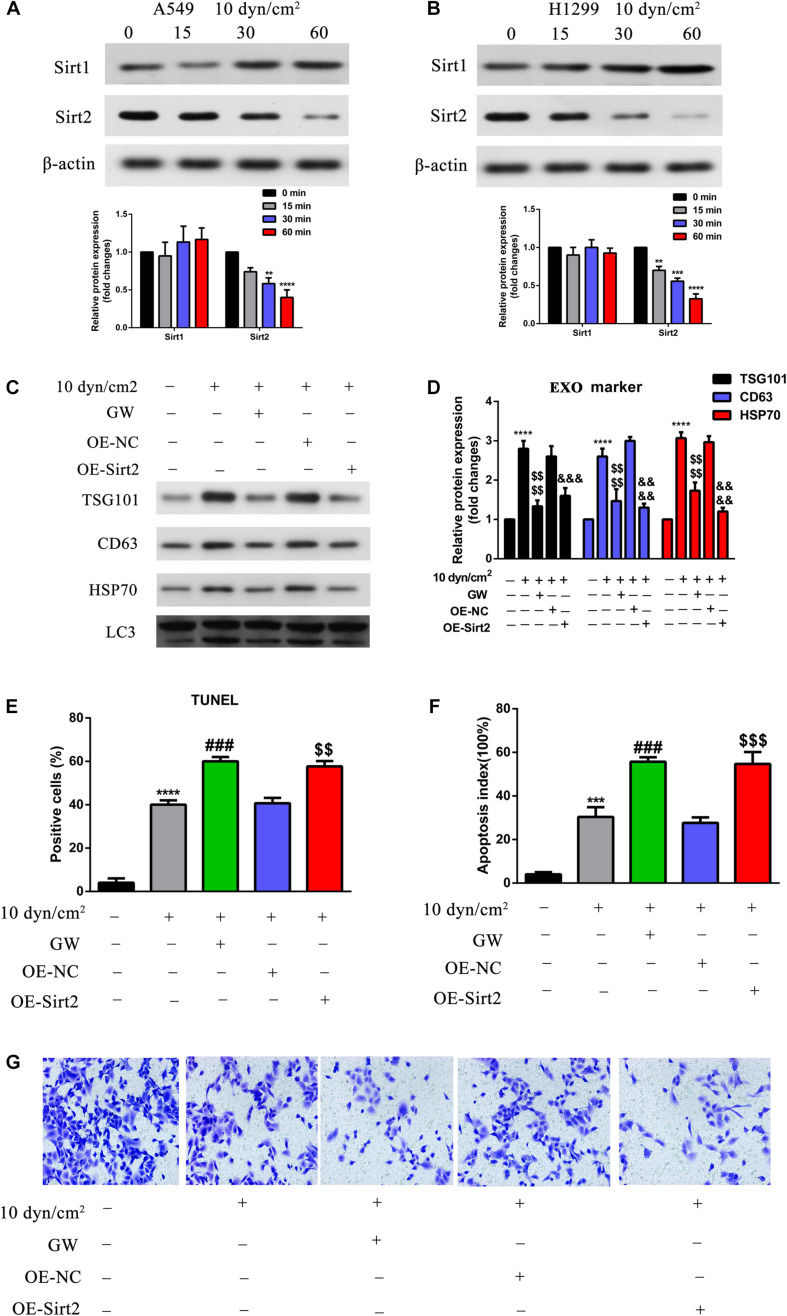
Over expression of SIRT2 reduced exosomes and autophagy component release, promoted ASS induced cell apoptosis, inhibited cell invasion. **(A,B)** A549 and H1299 cells were exposed to ASS (10 dyn/cm^2^) for indicated times, and then the expression of SIRT1 and SIRT2 were detected by western blot, each experiment was repeated at least three times, **P < 0.01, ***P < 0.001, ****P < 0.0001. **(C,D)** After overexpression of SIRT2 or treatment with GW4689, A549 cells were exposed to ASS (10 dyn/cm^2^) for 60 min, quantitative analysis of the released exosome (TSG101, CD63, HSP70) and autophagy component (LC3) markers were carried out by western blot, ****P < 0.0001 compared to control group, $$$$ denote *P* < 0.0001 compared to ASS (10 dyn/cm^2^) group, &&&, &&&& denote *P* < 0.001 and *P* < 0.0001 compared to OE-NC group. **(E,F)** Cell apoptosis rate were, respectively, examined by **(E)** TUNEL assay, and **(F)** flow cytometry after the cells were stably overexpressed for SIRT2 or pretreated with GW4689 for 60 min. Further, the cells were exposed to ASS for 60 min, *** and **** denote *P* < 0.001 and *P* < 0.0001 compared to control group,^###^ denote *P* < 0.001 compared to ASS (10 dyn/cm^2^) group, $$, $$$ denote **P < 0.01 and *P* < 0.001 compared to OE-NC group. **(G)** After overexpression of SIRT2 or treatment with GW4689, A549 cells were exposed to ASS (10 dyn/cm^2^) for 60 min, cell invasion was detected by Tranwell assay.

Previously, studies indicated the role of SIRT1 in exosome biogenesis. We were therefore interested to understand the roles of SIRT1 and SIRT2 in ASS associated autophagy and apoptosis. Initially, we performed western blotting analysis of ASS exposed A549 and H1299 cell lysates. It was clear that the increase in ASS exposure did not significantly affect the expression of SIRT1, while the expression of SIRT2 decreased significantly (Figures 4A,B). As stated earlier, exposure to ASS increased exosome release, apoptosis and decreased SIRT2 expression levels. Hence, we conducted experiments to assess the function of SIRT2 in exosome release and apoptosis. We overexpressed SIRT2 in A549 cells and observed a decrease in expression of exosome and autophagic markers after exposure to ASS for 60 min, indicating a decrease in the secretion of exosomes and autophagic components (Figures 4C,D). These data provided evidence to link our findings that SIRT2 is a negative regulator of the secretion of exosomes and autophagic components. In addition, to assess the impact of ASS and SIRT2 overexpression on apoptosis, we produced A549 cells that were stably overexpressing SIRT2 and performed TUNEL assay and flow cytometric analysis after exposure to ASS. Interestingly, both the TUNEL (Figure 4E) and flow cytometric assays (Figure 4F) showed that the overexpression of SIRT2 and ASS exposure increased apoptosis, higher than that in the ASS exposure only group. Another key observation was that after exposure to ASS, SIRT2 overexpressed cells mimicked the group of cells which were treated with GW (exosome inhibitor). At the same time, the Transwell assay (Figure 4G) revealed that SIRT2 overexpression and ASS exposure can significantly inhibit A549 cell invasion. This indicated that SIRT2 overexpression inhibited cell invasion and decreased secretion of exosome and autophagy factors thus increasing apoptosis in ASS exposed cells.

### TFEB Overexpression Regulated Cell Apoptosis, Autophagy, and Invasion

**FIGURE 5:**
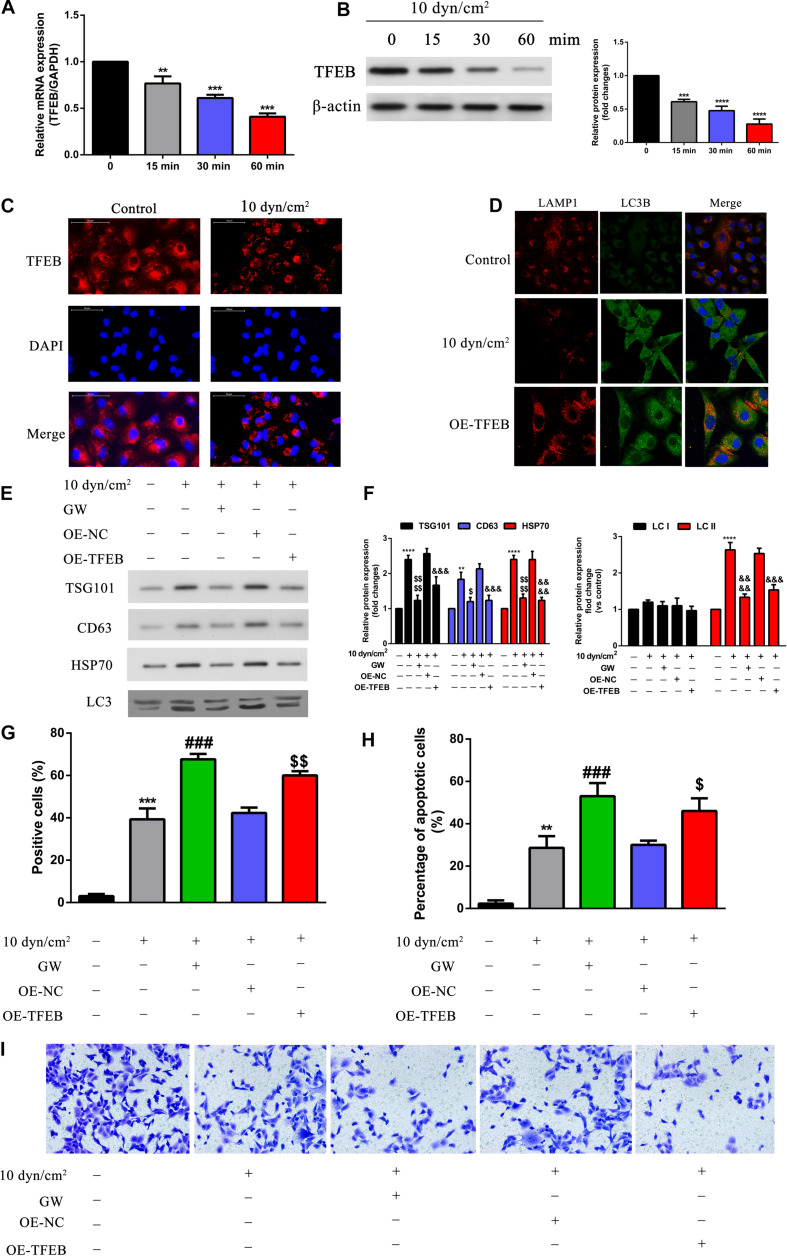
Overexpression of TFEB accelerated fusion of autophagosome and lysosome, inhibited exosomes and autophagy component release, promoted cell apoptosis, regulated cell invasion. **(A,B)** A549 cells exposed to ASS (10 dyn/cm^2^) for 0, 15, 30, and 60 min, the expression of TFEB was detected by qRT-PCR and western blot, **P < 0.01, ***P < 0.001, ****P < 0.0001. **(C)** The expression level of TFEB after exposure to ASS (10 dyn/cm^2^) for 60 min were confirmed by cell immunofluorescence. **(D)** Immunofluorescence of LAMP1 and LC3B was performed on A549 cells exposed to ASS (10 dyn/cm^2^) for 60 min, the co-localization of LAMP1 puncta and LC3B puncta was observed in the merge image (yellow merge area), magnification, 600×. **(E,F)** A549 cells, after stable overexpression of TFEB or GW4689 treatment, were exposed to ASS (10 dyn/cm^2^) for 60 min, then isolated exosomes and autophagic components were identified and quantified by western blot, ****P < 0.0001 compared to control group, $, $$$$ *P* < 0.05 and *P* < 0.0001 compared to ASS (10 dyn/cm^2^) group, &&&, &&&& denote *P* < 0.001 and *P* < 0.0001 compared to OE-NC group. **(G,H)** Cell apoptosis was determined by TUNEL assay and flow cytometry, each group was treated the same as panel E, ** and *** denote *P* < 0.01 and *P* < 0.001 compared to control group, ^###^*P* < 0.001 compared to ASS (10 dyn/cm^2^) group, $, $$$ denote *P* < 0.05 and *P* < 0.01 compared to OE-NC group. **(I)** A549 cells, after stable overexpression of TFEB or GW4689 treatment, were exposed to ASS (10 dyn/cm^2^) for 60 min, Tranwell assay was used to detect cell invasion.

Further, to understand how SIRT2 regulates autophagy, we assessed a downstream target of SIRT2, TFEB (transcription factor EB). A549 cells were exposed to ASS (10 dyn/cm^2^) for 0, 15, 30, and 60 min, and assessed for TFEB expression using qRT-PCR and western blotting. It was clear that ASS exposure decreased TFEB expression after 15 min, and both mRNA and protein TFEB levels decreased tremendously after 60 min (Figures 5A,B). Similarly, we also performed IFC staining and observed clear TFEB cytoplasmic staining in the control cells, whereas ASS exposed cells displayed decreased TFEB staining (Figure 5C). Previously, studies have indicated TFEB’s role in lysosomal biogenesis and autophagy (Sardiello et al., 2009; Napolitano and Ballabio, 2016). Hence, with the aid of A549 cells overexpressing TFEB, we performed IFC of key autophagic markers such as LAMP1 and LC3B after exposure to ASS (10 dyn/cm^2^). Evidently, ASS exposure clearly decreased LAMP1 expression, whereas TFEB overexpression rescued this inhibition of LAMP1 expression by ASS. Alternatively, ASS exposure increased LC3B expression but TFEB overexpression decreased its expression. Further, we could observe a strong co-localization of LC3B and LAMP1 specifically near the nuclear region in the TFEB overexpressed cell line, after exposure to ASS, indicating a potential increase in the fusion of autophagosome and lysosome (Figure 5D). Additionally, using western blotting, we observed that TFEB overexpression decreased exosome and autophagic components secretion after ASS exposure (Figures 5E,F). Using TUNEL assay and flow cytometry, we could confirm that TFEB overexpression increased apoptosis when compared to the control cells and thus mimicked the cells treated with GW (exosome inhibitor), after exposure to ASS (Figures 5G,H). In addition, through Tranwell assay (Figure 5I), we concluded that compared with the control cells, TFEB overexpression cells and the group of cells which were treated with GW significantly inhibited cell invasion, after exposure to ASS. These data indicated that TFEB overexpression decreased secretion of exosomes along with autophagic factors, accelerated autophagosome, and lysosome fusion, and increased apoptosis in ASS exposed NSCLC cells.

### SIRT2 Directly Binds and Regulates TFEB Expression

**FIGURE 6:**
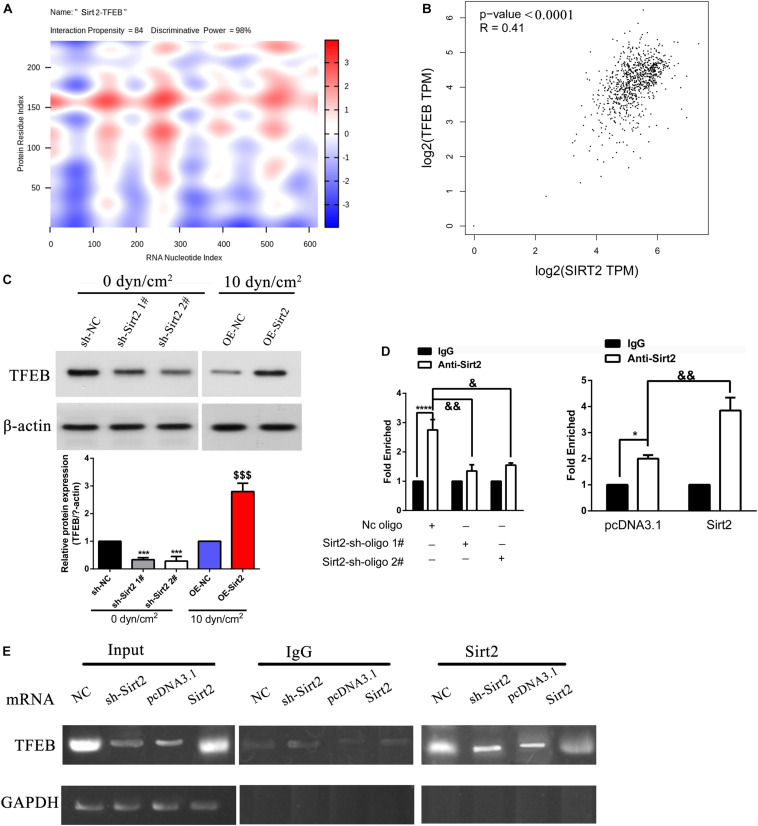
SIRT2 promoted TFEB expression through direct binding with TFEB mRNA. **(A)** Prediction of the interaction between SIRT2 and TFEB 3′ UTR indicated as a heat-map, *cat*RAPID identified the interaction between SIRT2 and TFEB with confidence (Interaction Propensity = 84, Discriminative Power = 98%). **(B)** Based on data from TCGA, Pearson correlation of SIRT2 and TFEB (*P* < 0.0001, *R* = 0.41). **(C)** A549 cells were either stably knocked-down or overexpressed for SIRT2, and then exposed to ASS (0 and 10 dyn/cm^2^) for 60 min, the expression of TFEB was determined by western blot, ***P < 0.001 compared to sh-NC group, $$$ *P* < 0.001 compared to OE-NC group. **(D,E)** Cellular extracts of NSCLC cells with either stable knockdown or overexpression of SIRT2 were immunoprecipitated with SIRT2 and IgG antibodies, the target TFEB mRNA of the immunoprecipitation was assessed through qRT-PCR and semi-quantitative RT-PCR. *P < 0.05, ****P < 0.0001.

Initially, using *cat*RAPID we assessed the interaction between SIRT2 and 3′ UTR of TEFB. Evidentially, heatmap analysis indicated that SIRT2 and TFEB 3′ UTR associated with a high interaction propensity score of 84. The software also validated that this interaction had a high predictability rate due to the high discriminative power score of 98% (Figure 6A). Based on NSCLC tissue samples from TCGA database, we further performed Pearson’s correlation analysis to assess the association between SIRT2 and TFEB. Incidentally, SIRT2 and TFEB shared a positive relationship with an *R*-value of 0.41 (*P* < 0.0001) (Figure 6B). To further assess the effect of SIRT2 on TFEB, SIRT2 was either overexpressed (OE) or silenced (sh) in A549 cells and exposed to ASS (10 dyn/cm^2^) for 60 min. Further, we performed western blotting analysis to assess the levels of TFEB expression. Silencing of SIRT2 in the static cells, significantly decreased TFEB expression levels similar to the expression in cells exposed to ASS (10 dyn/cm^2^). However, overexpression of SIRT2, significantly recovered the TFEB expression levels after exposure to ASS (Figure 6C). This indicated that TFEB expression levels were SIRT2 dependent. RNA immunoprecipitation studies were also performed to confirm the interaction between SIRT2 and TFEB. Initially, A549 cells were either silenced or overexpressed for SIRT2 expression and after 48 h, we subsequently performed immunoprecipitation experiments on cell lysates using SIRT2 and control igG antibodies. Further, from the immunoprecipitated samples, the levels of target mRNA were assessed through qRT-PCR and semi-quantitative PCR. For the SIRT2 silenced cells, we identified significantly low levels of TFEB mRNA, however, for the cells which were overexpressed for SIRT2, we identified high levels of TFEB mRNA (Figures 6D,E). These evidences clearly indicated that SIRT2 promoted TFEB expression through direct binding with TFEB mRNA.

### SIRT2 Along With TFEB Regulates Autolysosome Formation, Exosomes, and Autophagy Component Release, Cell Apoptosis, and Invasion

**FIGURE 7:**
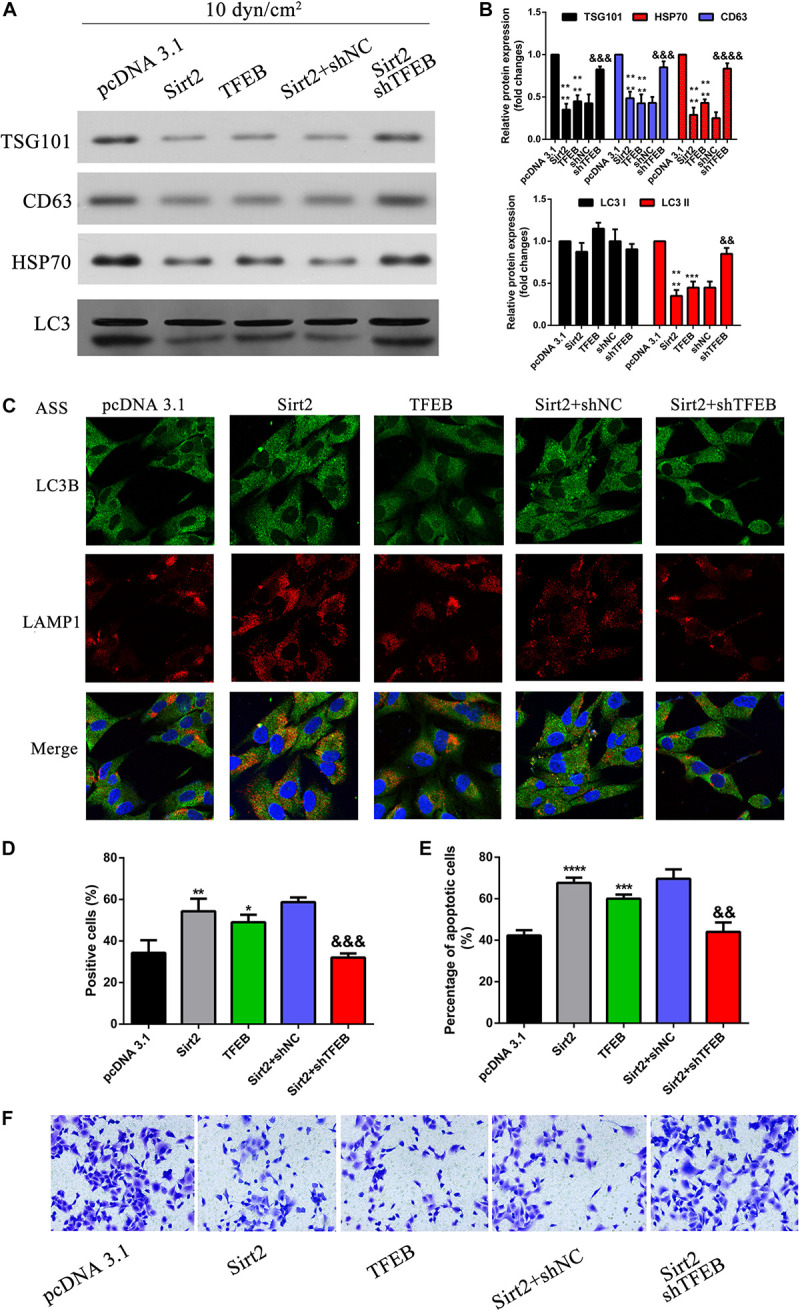
SIRT2 along with TFEB regulates autolysosome formation, exosomes, autophagy component release, cell apoptosis, and invasion. **(A,B)** NSCLC cells transfected with OE-SIRT2, OE-TFEB, or OE-SIRT2/sh-TFEB plasmid were exposed to ASS (10 dyn/cm^2^) for 60 min, exosomes which were released were collected and exosome specific markers were detected by western blot, ****P < 0.0001 compared to pcDNA3.1 group, &&&, &&&& denote *P* < 0.001 and *P* < 0.0001 compared to shNC group. **(C)** After treatment as previously described, immunofluorescence was performed to detect the fluorescence intensity of LAMP1 and LC3B, the co-localization of LAMP1 and LC3B was observed as yellow region in the merged image, magnification, 600×. **(D,E)** The percentage of cell apoptosis was determined by TUNEL assay and flow cytometry, *, **, *** and **** denotes *P* < 0.05, *P* < 0.01, *P* < 0.001, and *P* < 0.0001 compared to pcDNA3.1 group, &&, &&& denote *P* < 0.01 and *P* < 0.001 compared to SIRT2/shNC group. **(F)** After treatment as previously described, cell invasion was measured by Tranwell assay.

To understand the role of TFEB and its association with autophagy, A549 cells were overexpressed for SIRT2 and silenced for TFEB and exposed to ASS for 60 min. As observed previously, overexpression of SIRT2 or TFEB, significantly decreased secretion of exosomes and autophagy factors, as indicated by western blotting analysis of TSG101, CD63, HSP70, and LC3 (Figures 7A,B). However, when SIRT2 was overexpressed but TFEB was silenced, we observed an opposite effect. This indicated that the effect of SIRT2 on secretion of autophagic factors and exosomes were dependent on TFEB expression. Further, using IFC, we could observe that SIRT2 or TFEB overexpression increased LAMP1 expression and co-localization with LC3B. However, when SIRT2 was overexpressed but TFEB was silenced, the expression of LAMP1 significantly decreased along with decrease in co-localization of LAMP1 and LC3B, indicating its effect on autolysosome formation (Figure 7C). Subsequently, we checked TFEB’s effect on apoptosis using flow cytometry and TUNEL assay. Similar to the previous evidence, overexpression of SIRT2 and silencing of TFEB, significantly decreased the number of apoptotic cells compared to the SIRT2 or TFEB overexpressing cells (Figures 7D,E). At the same time, Transwell assay (Figure 7F) proved overexpression of SIRT2 and silencing of TFEB, significantly increased cell invasion compared to the SIRT2 or TFEB overexpressing cells. These evidences clearly indicate that TFEB is required for SIRT2’s effect on autolysosome formation, exosomes, and autophagy component release, cell apoptosis, and regulated invasion in NSCLCs.

### SIRT2 Overexpression Inhibited NSCLC Cell Metastasis *in vivo*

**FIGURE 8:**
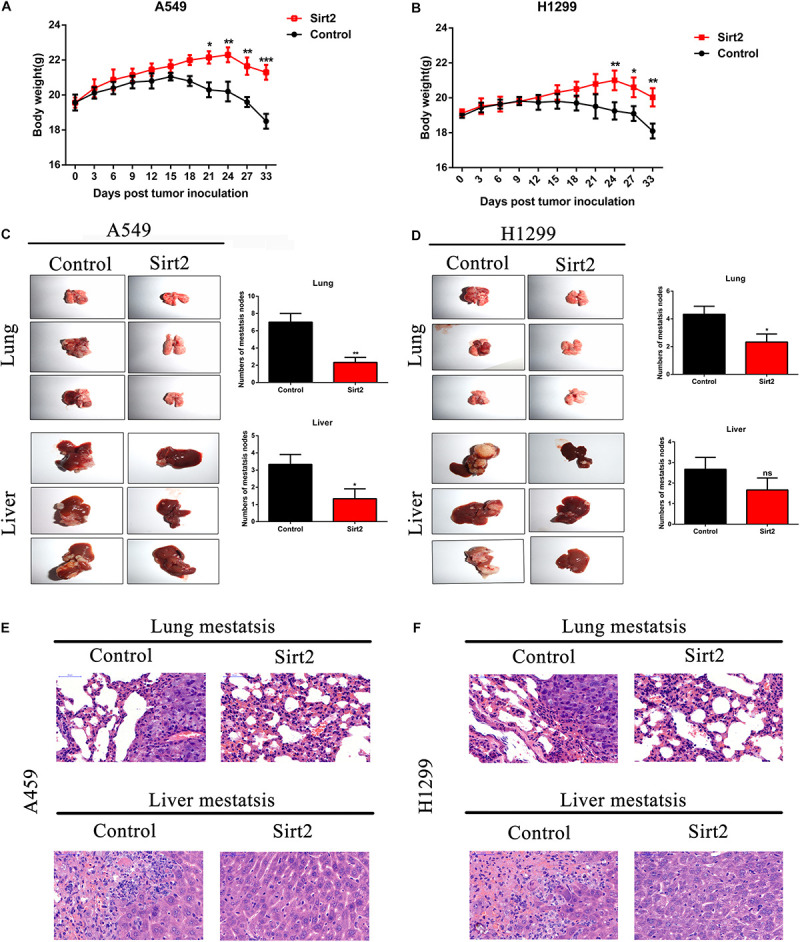
SIRT2 overexpression inhibited NSCLC cell metastasis *in vivo*. **(A,B)** SIRT2 overexpressing A549 cells or H1299 cells were injected into nude mice, the weight of each mouse were detected for every 3 days, after 33 days, the mice were sacrificed and liver and lung metastasis nodules were observed and imaged (*n* = 6 mice per group, *P < 0.05, **P < 0.01, ***P < 0.001). **(C,D)** Six weeks after injection with SIRT2 over-expressed A549, H1299, or control cells, representative photos of nude mouse metastatic nodules of lung and liver were imaged. Black arrowheads show metastatic nodules in lungs and livers and the total numbers of metastatic nodules were assessed (*n* = 6 mice per group, *P < 0.05, **P < 0.01). **(E,F)** Representative images of hematoxylin and eosin staining of lung and liver metastasis nodule, Scale bar, 50 μm.

To assess the role of SIRT2 *in vivo* NSCLC cells (A549 and H1299) were transfected with SIRT2 overexpression systems, and were injected into nude mice (*n* = 6 mice per group). Mice were monitored and weighed once every 3 days for 33 days. Compared to the mice injected with NSCLC control cells (A549, H1299; *n* = 6 mice per group), nude mice injected with SIRT2 overexpressing cells had lesser weight loss (Figures 8A,B). After 33 days, mice were sacrificed and images were taken of the lung and liver tissues to assess the formation of metastatic nodules (Figures 8C,D). Evidentially, SIRT2 over-expression significantly decreased metastatic nodule formation in the mice lung and liver tissues. We subsequently performed hematoxylin and eosin staining of these tissue sections and observed severe metastasis in the NSCLC injected mice models (Figures 8E,F). However, mice injected with SIRT2 over-expressing cells showed significantly less metastasis compared to the mice injected with control cells.

### SIRT2 and TFEB Are Down-Regulated in Human NSCLC Tissues and Positively Correlated With Overall Survival in TCGA Database

**FIGURE 9:**
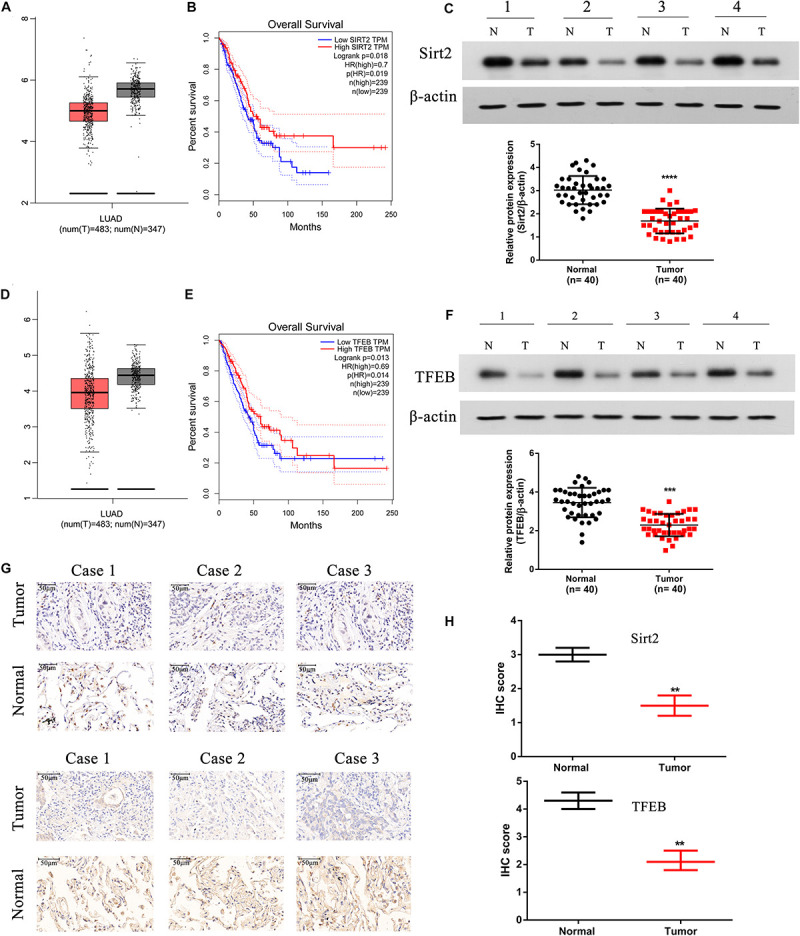
SIRT2 and TFEB are down-regulated in human NSCLC tissues and positively correlated with overall survival in TCGA database. **(A,B)** The expression of SIRT2 in lung adenocarcinoma (LUAD) patients (*t* = 483, *n* = 347) and the overall survival (OS) curves of SIRT2^high^ (*n* = 239) and SIRT2^low^ (*n* = 239) NSCLC patients. **(C)** Total protein was extracted from human NSCLC tissues (*n* = 40), SIRT2 was detected by western blot, the upper panel shows SIRT2 expression in four tumor tissues samples with corresponding normal lung tissues samples. And the lower panel indicates the quantification of SIRT2 protein level in NSCLC tissues and corresponding normal lung tissues (*n* = 40, ****P < 0.0001). **(D,E)** The expression of TFEB in LUAD patients (*t* = 483, *n* = 347) and the OS curves of TFEB^high^ (*n* = 239) and TFEB^low^ (*n* = 239) NSCLC patients. **(F)** The expression of TFEB was detected in human NSCLC tissues, the upper panel shows the expression of TFEB in four tumor tissues samples with corresponding normal lung tissues samples. And the lower panel indicates the quantification of SIRT2 protein level in NSCLC tissues and corresponding normal lung tissues (*n* = 40, ***P < 0.001). **(G,H)** IHC staining detected the expression of SIRT2 and TFEB in human NSCLC tissues (*n* = 40, **P < 0.01).

We assessed the expression levels of SIRT2 and TFEB in the TCGA-LUAD database, and observed that both SIRT2 and TFEB were highly expressed in the LUAD patients (Figures 9A,D). Further, from the database, two groups were identified based on the SIRT2 and TFEB expression levels. It was evident that increased levels of SIRT2 and TFEB were associated with high survival rate (Figures 9B,E). Further, we performed western blotting analysis of the protein samples from patient NSCLC lung tissue and normal lung tissue samples, and observed that NSCLC tissues had significantly lower expression of SIRT2 and TFEB levels (Figures 9C,F). Additionally, we also performed IHC staining of the tissue sections and observed significantly low levels of both SIRT2 and TFEB levels in the NSCLC tumor tissues (Figures 9G,H). These evidences clearly indicate that SIRT2 and TFEB levels are significantly down-regulated in NSCLC patients and low expression levels of these transcripts are associated with low-survival rates.

## Discussion

Exposure to various mechanical stress such as fluid SS (FSS), low SS (LSS), and ASS instigates multiple responses in different cell types. In endothelial cells, SS is necessary for the organization and functional activity of the cells (Girard and Nerem, 1993). However, in cancer cells SS initiates a cascade of protective mechanisms which allows them to migrate, invade, and proliferate in target regions, which is a key process to establish metastasis in cancer. Based on previous studies, LSS (5–30 dyn/cm^2^) seem to increase migration and invasion of the cancer cells whereas high stress (160 dyn/cm^2^) leads to tumor cell death. In our study, use of ASS (10 dyn/cm^2^) for 60 min led to a significant increase in migration and invasion of NSCLC cells in our *in vivo* models as indicated by increased metastatic nodules in liver and lung tissues of our nude mice model (Figure 8B). Previously, a study by Aung et al. (2014) showed that low level 3D stress on a breast cancer lines increased its migration and invasion in a protease dependent manner. In another study in hepatocellular carcinoma (HCC), it was indicated that this increase in migration and invasion could be through the rearrangement of actin cytoskeleton and integrin signaling which also plays a key role in autophagy (Zeng and Tarbell, 2014). In starved HeLa cells, microfilament and autophagic markers were colocalized and inhibition of microfilaments decreased autophagy as well (Aguilera et al., 2012). Further another study on HepG32 cells, confirmed the role of SS on autophagy and cytoskeletal proteins as FSS seems to induce cytoskeletal remodeling and migration through activation of autophagy (Yan et al., 2019). In our study, we found a significant increase LC3B puncta after increased exposure to ASS (10 dyn/cm^2^) for 60 min (Figures 1A–D). Further, we observed an increase in secretion of exosomes containing autophagy factors such as LC3B after exposure to ASS (Figures 2F–K). Another study indicated that ASS induced formation, accumulation, and fusion of autophagosome with multivesicular body which is then released into the extracellular space through EVs (Wang et al., 2019). We also observed that exposure to ASS increased apoptosis in NSCLCs, however, inhibition of exosome release significantly increased apoptotic rate (Figures 3C–F). These results clearly indicated that the cancer cells releases Exosomes with the autophagic components as a protective mechanism against apoptosis.

To understand the mechanism underlying such release in exosomes and autophagic components by NSCLC exposed to ASS, we explored the role of sirtuins, a family of proteins previously identified to be associated with autophagy and lysosomal pathway. A study by Latifkar et al. (2019) (McAndrews et al., 2019), identified sirtuin 1 (SIRT1) levels were significantly decreased in the triple negative breast cancer patients, and from their *in vitro* studies it was evident that the knockdown of SIRT1 significantly increased release of exosomes and autophagic factors. In our study, interestingly we observed no significant decrease in SIRT1 levels, however, we observed a significant decrease in SIRT2 levels in NSCLC cells exposed to ASS (Figures 4A,B). Further, overexpression (OE) of SIRT2 significantly decreased exosome and autophagic component release, this effect was similar to the use of autophagic inhibitor (GW4689) (Figure 4C). Additionally, it was also evident that OE of SIRT2 increased apoptosis of NSCLCs (Figure 4E), thus clearly indicating that SIRT2 is necessary for the anti-tumorigenic effect of ASS.

Next, we were curious as to how SIRT2 regulated autophagy in NSCLCs, and assessed the expression of its down stream target TFEB. In NSCLCs, we observed that TFEB was also highly downregulated, and silencing of SIRT2 decreases TFEB expression (Figure 6C). RNA immunoprecipitation assays indicated that SIRT2 binds to the 3′ UTR of TFEB and thus regulates its expression (Figure 6E). TFEB has been previously reported to link autophagy and lysosomal biogenesis pathways (Settembre et al., 2011). Specifically, TFEB seems to be phosphorylated by mTOR and the dephosphorylated TFEB migrates to the nucleus where it activates many lysosomal and autophagy related target genes (Napolitano and Ballabio, 2016). Further, TFEB also has been identified to bind to many autophagy associated genes and thus activate autophagosome biogenesis and autophagosome–lysosome fusion (Settembre et al., 2011). In the current study, we observed that TFEB overexpression increased the autophagosome and lysosome fusion as indicated by the increased co-localization of LC3B and LAMP1 near the nucleus. It was also evident that TFEB overexpression decreased exosomes, autophagic component release, and increased apoptosis in ASS exposed NSCLC cells (Figure 7).

We also observed that SIRT2 overexpression significantly decreased the levels of metastatic nodules in the lungs and liver of the *in vivo* mice model (Figures 8C–F). Further, we observed that SIRT2 and TFEB were downregulated in human NSCLC tissues and their decreased expression correlated with poor prognosis in patients (Figure 9).

## Conclusion

These evidences clearly indicate for the first time, the role of SIRT2 and TFEB in tumorigenesis of NSCLCs. Further, it also sheds light on the protective mechanisms of cancerous cells under exposure to ASS, thus providing potential drug targets for the treatment of NSCLC. Further, studies on LSS in NSCLC could shed more light on the intrinsic mechanisms associated with metastasis and tumorigenesis.

## Data Availability Statement

The original contributions presented in the study are included in the article/supplementary material, further inquiries can be directed to the corresponding author/s.

## Ethics Statement

The animal study was reviewed and approved by the Ethical Committee for Animal Research of Xin Hua Hospital Affiliated to Shanghai Jiao Tong University School of Medicine. Written informed consent was obtained from the individual(s), and minor(s)’ legal guardian/next of kin, for the publication of any potentially identifiable images or data included in this article.

## Author Contributions

HX and HZ conceived the project and designed the research. LW, PX, XX, FH, LJ, RH, and FD performed the experiments. LW, PX, HX, and HZ analyzed the data, discussed the research, and wrote the manuscript. All authors contributed to the article and approved the submitted version.

## Conflict of Interest

The authors declare that the research was conducted in the absence of any commercial or financial relationships that could be construed as a potential conflict of interest.

## Abbreviations

ASS: acute shear stress
Exo: exosome
NSCLC: non-small lung cancer
SIRT2: sirtuin 2
TFEB: transcription factor EB.
